# Update on the Management of Diabetic Retinopathy: Anti-VEGF Agents for the Prevention of Complications and Progression of Nonproliferative and Proliferative Retinopathy

**DOI:** 10.3390/life13051098

**Published:** 2023-04-27

**Authors:** Tyler A. Bahr, Sophie J. Bakri

**Affiliations:** Mayo Clinic, Department of Ophthalmology, 200 First St SW, Rochester, MN 55902, USA

**Keywords:** diabetic retinopathy, vascular endothelial growth factor, VEGF, anti-VEGF, intravitreal injection, eye

## Abstract

Diabetic retinopathy (DR) is a microvascular disease caused by poorly controlled blood glucose, and it is a leading cause of vision loss in people with diabetes. In this review we discuss the current management of DR with particular focus on the use of intraocular anti-vascular endothelial growth factor (anti-VEGF) agents. Intraocular anti-VEGF agents were first studied in the 1990s, and now several of these agents are either FDA approved or used off-label as first-line treatments for DR. Recent evidence shows that anti-VEGF agents can halt the progression of markers of DR severity, reduce the risk of DR worsening, and reduce the onset of new macular edema. These significant benefits have been demonstrated in patients with proliferative DR and the milder nonproliferative DR (NPDR). A wealth of evidence from recent trials and meta-analyses has detailed the intraoperative and postoperative benefits of adjunctive anti-VEGF therapy prior to pars plana vitrectomy (PPV) for proliferative DR with vitreous hemorrhage. In this review, we also discuss literature comparing various anti-VEGF injection regimens including monthly, quarterly, as-needed, and treat and extend protocols. Combination protocols with panretinal photocoagulation (PRP) or PPV are also discussed. Current evidence suggests that anti-VEGF therapies are effective therapy for NPDR and PDR and may also provide significant benefits when used adjunctively with other DR treatment modalities such as PRP or PPV.

## 1. Introduction

Diabetic retinopathy (DR) is a significant public health concern as the leading cause of vision loss in people with diabetes. DR can cause permanent, disabling vision loss that affects either peripheral or central vision. Approximately one-third of all diabetics have some form of retinopathy, with over 100 million cases of DR worldwide. Of the diabetics with DR, about one-third have a vision-threatening form of the disease. By 2045 the prevalence of DR is expected to increase by more than 50% [1].

The pathogenesis of DR is complex and involves several mechanisms. An emerging hypothesis is that in the early stages of DR, retinal neurodegeneration may play a major role as an inciting factor, prior to microvascular changes. Downregulation of retinal neurotrophic factors and neuron loss in early DR are thought to be linked with processes such as glutamate excitotoxicity, oxidative stress, neuroinflammation, and renin-angiotensin system overactivation. Laboratory studies have linked these pathways with derangements in angiogenic factors which are known to be central in DR pathogenesis [2]. 

With prolonged elevated blood glucose, the accumulation of glycation end products (damaged protein) and other deposits leads to wall thickening and endothelial injury in the small vessels of the retina. This process is worsened by comorbid hypertension and hyperlipidemia. Over time, these vascular changes result in tissue ischemia and pathologic vascular remodeling, blood-retinal barrier breakdown, and increased vascular permeability, allowing for leakage of protein and fluids into the neuroretinal layers. In turn, this can cause focal edematous changes, hemorrhages, and visible vascular abnormalities. Meanwhile, ischemic retinal tissue triggers an inflammatory and angiogenic cascade, with upregulation of the vascular endothelial growth factor (VEGF) and inflammatory cytokines leading to pathologic neovascularization and fibrous tissue formation. This only worsens the retinopathy and, if unchecked, can cause profound visual loss.

Nonproliferative diabetic retinopathy (NPDR) is defined as changes in the retinal vasculature without pre-retinal neovascular disease. These changes can include retinal hemorrhages, microaneurysms, changes in venous caliber, and intraretinal microvascular abnormalities. Proliferative diabetic retinopathy (PDR) is a more severe stage of DR and is characterized by pre-retinal or other ocular neovascularization. This neovascularization can consist of neovascularization of the disc (NVD), neovasculari zation elsewhere (NVE), or neovascularization of the iris (NVI), or a combination of these. Diabetic macular edema (DME) is a severely vision-threatening complication of DR. It is the most common cause of vision loss in patients with DR [3], and its onset can occur during any stage of the disease. 

In this review, we focus on current treatment of NPDR and PDR, acknowledging the treatment of DME as a relevant but broad topic of research which will not be discussed in this article. Here, we will review recent reports on anti-VEGF efficacy in different clinical scenarios for the treatment of nonproliferative and proliferative diabetic retinopathy, compare anti-VEGF versus traditional DR therapies, and discuss the benefits of adjunctive combinations. 

## 2. Overview of Treatments for DR

Currently there are several available treatments for DR, and each is focused primarily on targeting abnormal retinal vessels and/or angiogenesis. However, each has a distinct mechanism of action. For the past 40 years, panretinal photocoagulation laser therapy has been the standard of care for PDR and involves the placement of many (over 1000) laser spots in the peripheral retina resulting in ablation of ischemic peripheral retinal tissue to reduce VEGF production. This tissue destruction comes with some visual side effects including temporary visual disturbances such as glare or halos around lights, difficulty with night vision and, in some cases, patients may also experience permanent peripheral visual field loss. The Diabetic Retinopathy Study (DRS) [4] demonstrated that panretinal photocoagulation (PRP) reduced the two-year incidence of severe vision loss by 60%, and the Early Treatment of Diabetic Retinopathy Studies (ETDRS) showed a 45% relative risk reduction for early compared with late PRP [5].

Pars plana vitrectomy (PPV) is a surgical procedure for the treatment of DR with significant vision-threatening complications such as vitreous hemorrhage or tractional retinal detachment. PPV involves the removal of the vitreous gel from the eye, and it is often performed in combination with other procedures such as endolaser, gas or oil tamponade.

Anti-VEGF therapies are a newer option for the treatment of DR, and an accumulating body of recent evidence suggests they are effective in slowing the progression of DR. These therapies work by inhibiting the action of VEGF, a signaling factor that promotes angiogenesis. Anti-VEGF therapy for DR is administered via intravitreal injection, which requires frequent injections, as often as monthly. Other methods for delivering anti-VEGF agents to the vitreous, such as implantable devices and gene delivery vectors, are actively under investigation in clinical trials.

## 3. Anti-VEGF Agents and Their Mechanisms

Anti-VEGF therapies were first developed in the 1990s to block the vascular endothelial growth factor, which plays a key role in angiogenesis. Initially, these therapies were used to treat cancer, but were found to be effective in treating ocular conditions, including age-related macular degeneration, macular edema, and DR. Today there are several anti-VEGF agents that are FDA approved for the treatment of DR (Table 1).

### 3.1. Off-Label Use of Anti-VEGF Therapy

Bevacizumab (Genentech, San Francisco, CA, USA) was the first FDA-approved VEGF inhibitor dating back to 2004, however, it was approved for the treatment of metastatic colorectal cancer, not for intraocular use. Since then, although trials have demonstrated its efficacy for various retinal diseases, it has been used off-label for intraocular injections for nearly two decades. Bevacizumab is a recombinant humanized monoclonal antibody of 149 kDa size, comprised of two mouse antibody binding regions targeting VEGF-A, with a truncated human IgG1 heavy chain [6].

### 3.2. Anti-VEGF Agents with FDA Approval for DR

Ranibizumab (Genentech, San Francisco, CA, USA) was originally FDA approved for exudative macular degeneration, and in 2017 was approved for the treatment of DR. Ranibizumab is a recombinant monoclonal antibody fragment with one VEGF-A binding site, created from the same mouse antibody as bevacizumab, but lacking the fragment crystallizable (Fc) region and only 48 kDa in size, meaning that it avoids the Fc recycling and can more easily penetrate retinal tissue [6]. 

Aflibercept (Regeneron, Tarrytown, NY, USA) was also originally approved for exudative macular degeneration, and in 2019 was FDA approved for DR. Aflibercept is a soluble decoy receptor with greater affinity than the natural receptors (a recombinant fusion protein of size 115 kDa consisting of two VEGF-binding domains, one each from the VEGF-1 and VEGF-2 receptors) fused to the Fc domain of IgG1. It traps VEGF-A, VEGF-B, and PIGF and directs them for consumption and degradation by phagocytes. It may also inhibit the action of placental growth factor (PIGF), which is also associated with angiogenesis and neovascularization. Because VEGF naturally occurs as a dimer, aflibercept binds two VEGF molecules simultaneously in its two sites, creating a very high affinity interaction [6]. 

### 3.3. Anti-VEGF Agents with FDA Approval for Other Ocular Conditions

Brolucizumab (Novartis, Cambridge, MA, USA) was approved for exudative age-related macular degeneration in 2019 and DME in 2022, however, it is not yet FDA approved for DR. Brolucizumab is a humanized monoclonal single-chain variable fragment of size 26 kDa that binds VEGF-A with a single binding site, however, brolucizumab:VEGF binding occurs in a 2:1 ratio [7]. Studies of brolucizumab for the treatment of DR are underway (NCT 04278417).

Faricimab (Roche, Basel, Switerzland) was FDA approved for wet AMD and DME in 2022, however, it has not yet been approved for DR. It is a dual-mechanism antibody of 149 kDa in size with two different antigen-binding fragment regions, one which targets VEGF and the other targeting Ang-2, connected to a single Fc domain [6]. Studies of Faricimab for the treatment of DR are underway (NCT 05681884).

## 4. Updates on the Safety and Adverse Effects of Intraocular Anti-VEGF Therapy

Intravitreal injection itself comes with risks such as endophthalmitis or transient intraocular pressure elevation, and this is well characterized. Apart from the risks of injection itself, several studies have investigated the safety and efficacy of intravitreal anti-VEGF injection. Various ocular adverse events associated with the intravitreal injection of these agents have been reported including retinal detachment, cataracts, endophthalmitis, elevated intraocular pressure, vitreous hemorrhage, uveitis and ocular inflammation, floaters, and retinal vessel changes [8]. There have also been concerns regarding the risk of glaucomatous optic neuropathy with long-term anti-VEGF therapy [9]. Additionally, anti-VEGF treatment may affect circulating VEGF levels, which has raised concern for the possibility of systemic adverse events. It has also been suggested that the risk of endophthalmitis may increase as the number of injections increase; given the frequency of injections in many anti-VEGF regimens, this potential increase of endophthalmitis risk merits consideration. 

A meta-analysis of 11 studies that focused on investigating systemic adverse events associated with Bevacizumab and Ranibizumab found no statistically significant increase in the risk of any adverse events, including overall mortality, arterial thromboembolic events, vascular death, stroke, myocardial infarction, hemorrhage, and hypertension, compared to control regimens. Bevacizumab did not appear to be associated with an increased risk of adverse events compared to ranibizumab, although there was a suggestion that ranibizumab may be associated with an increased risk of non-ocular hemorrhage in older patients with macular degeneration [10,11,12,13,14,15].

A more recent meta-analysis of 52 trials found that a higher number of injections was not associated with a significant increase in mortality risk at 12 or 24 months [16]. In a prospective study of 40 patients receiving monthly injections of either intravitreal bevacizumab or ranibizumab, there were no signs of cardiotoxicity as measured by cardiac troponin T levels and a decrease in plasma VEGF levels that correlated with the number of injections. However, the decrease in VEGF levels was only statistically significant in the bevacizumab group at Week 24 [17].

Concerns have been raised about the potential for anti-VEGF therapy to increase the risk of tractional retinal detachment in patients with PDR, which is especially concerning in patients prone to subsequent loss of follow-up [18,19]. However, a multicenter study that pooled data from 5 clinical trials involving a total of 487 eyes treated with anti-VEGF therapy and 396 eyes treated with other regimens found no increased risk of tractional retinal detachment associated with anti-VEGF therapy [20]. 

Thus, it seems that the risks of anti-VEGF therapy originate more from the injection itself rather than the agent. There is robust clinical data suggesting that intravitreal anti-VEGF agents are safe and effective, and there is no data to suggest an increase in mortality or adverse systemic events, adverse ocular events, or risk of retinal detachment, compared with sham injection. 

On the other hand, other therapies for the treatment of diabetic retinopathy including PRP and PPV have been associated with significant adverse effects. The peripheral tissue destruction by thermal burns in PRP can cause temporary visual disturbances such as glare or halos around lights, long-term problems with night vision and, in some cases, permanent peripheral visual field loss. Significant anatomic complications of PRP have also been reported including choroidal effusions, retinal detachments, and new onset macular edema [21]. Commonly reported complications of PPV include (but are not limited to) retinal detachment, suprachoroidal hemorrhage, endophthalmitis, and hypotony [22]. 

## 5. Efficacy of Anti-VEGF Agents as Primary Treatment for NPDR and PDR

### 5.1. Anti-VEGF as Primary Therapy for NPDR: Prevention of PDR, DME, and DR Worsening

Anti-VEGF agents have been shown to be effective as prophylaxis for worsening DR in patients with NPDR. DRCR Protocol W investigated the use of prophylactic aflibercept every four months in patients with moderate–severe NPDR for the prevention of DME and PDR. The study included 399 eyes and found that the two-year probability of developing CI-DME or PDR was 16.3% with aflibercept and 43.5% in the control group. However, the mean change in baseline visual acuity was not significant when comparing prophylactic aflibercept to sham [23]. Results from a four-year follow-up were similar, demonstrating some improvements in anatomic markers but no significant visual acuity benefit [24].

The PANORAMA trial, a double-blind randomized controlled trial (RCT), involved 402 adults with DRS level 47 or 53, no macular edema, and best corrected visual acuity (BCVA) of 20/40 or better, treated with either aflibercept every 16 weeks or every 8 weeks compared to sham. At 24 weeks, 58.4% of eyes treated with either regimen of aflibercept had a two-step improvement in DRSS scores compared to sham. At 52 weeks, 65% of the pooled aflibercept-treated groups had a two-step or greater improvement in DRSS scores compared to sham. Through Week 100, the rate of vision-threatening complications and CI-DME was significantly reduced compared to controls [25]. These results suggest that anti-VEGF agents such as aflibercept may be effective in reducing both the risk of DR worsening and also the onset of new DME in patients with NPDR.

A summary of the evidence for the uses and benefits of anti-VEGF agents across all stages of diabetic retinopathy is shown is Table 2.

### 5.2. Anti-VEGF as Primary Therapy for PDR: Prevention of PDR Progression

Anti-VEGF therapy may be useful for stopping the progression of PDR, as demonstrated in the RECOVERY trial, a prospective 12-month trial in 40 eyes of 40 patients with PDR and no DME, which investigated the effects of anti-VEGF therapy on the progression of PDR in patients randomized to either quarterly or monthly injections of 2 mg aflibercept. DRSS scores improved at least two steps in 67% and 74% of patients in the quarterly and monthly groups, respectively [26]. Overall, the mean total area of retinal nonperfusion in mm^2^ using UWF-FA imaging did not show a statistically significant change from baseline in the RECOVERY trial. There was not widespread evidence of retinal reperfusion with the aflibercept treatment, however, there was no progression of nonperfusion either. Mean vascular density showed no statistically significant difference from baseline after 12 months of treatment. However, the low statistical power of the study may have contributed to the lack of statistical significance from baseline [26].

In the second year of the trial, treatment crossover took place, in which subjects receiving monthly injections were now receiving quarterly injections (Arm 1) and vice versa (Arm 2). In all subjects, the ischemia index (the nonperfused area divided by the total retinal area visualized in the FA arteriovenous phase) increased from 25.8% to 50.4% while the mean retinal nonperfusion index (total area of retinal nonperfusion) increased from 235 mm^2^ to 402 mm^2^. DRSS scores were also significantly improved from baseline across all subjects, and no subjects experienced worsening of DRSS scores compared to baseline [27].

Results from the RECOVERY trial suggest that anti-VEGF therapy may be effective in reducing the progression of PDR, as demonstrated by improved DRSS scores and maintained retinal perfusion in individuals treated with monthly or quarterly aflibercept. Results from post hoc analyses of the same study suggest that anti-VEGF therapy prevented progression of other markers of DR severity, including nonperfusion index, microaneurysm count, leakage index, and retinal vascular bed area.

### 5.3. Treatment Planning

Although clinical trial data supports repeated intravitreal anti-VEGF injections for controlling PDR progression, the follow-up burden and cost of regular intraocular anti-VEGF injections is significant. Therefore, comparison of different regimens is needed. In addition to fixed-interval (i.e., monthly) dosing, other intravitreal injection regimens such as pro re nata (PRN) and treat and extend (T&E) methods have been considered, described in more detail below.

#### 5.3.1. Fixed-Interval Regimens: Monthly versus Quarterly

The RECOVERY trial compared monthly and quarterly aflibercept injection regimens in patients with PDR. At the six-month visit, the results showed that there was a significant improvement in the DRSS score in the monthly treatment group compared to the quarterly group [28]. The monthly, but not quarterly group, achieved a statistically significant decline in microaneurysm count and leakage index (the area of leakage on UWF-FA divided by the total visualized retinal area) by six months compared to baseline [29]. Retinal bed vascular area (RBVA, calculated as the total area of occupied retinal vessels divided by the total analyzable retinal area) decreased significantly compared to baseline in both groups by six months, however, the magnitude of RBVA decrease was roughly 50% in patients receiving monthly injections, but only 25% in patients receiving quarterly injections [30].

At one year, comparison of perfusion area between the monthly cohort and the quarterly cohort was statistically significant, with monthly dosing maintaining a stable perfusion area, compared with quarterly which demonstrated an increase in approximately 60 sq mm on average [26]. There was no statistically significant difference in DRSS score, VFQ-25, or VFQ-39 scores between the two groups at one year [28]. Although it took place later than the monthly group, by one year the quarterly group was also able to achieve a statistically significant decline in microaneurysm count and leakage index compared to baseline [29,31].

Crossover from quarterly to monthly and vice versa took place at the end of Year One. At the end of Year Two, 81% of patients who received monthly treatment in the second year had improvement of at least two steps in the DRSS score, compared with 65% of patients who received quarterly treatment in the second year [27].

These results indicate that by most measures, quarterly injections yielded comparable outcomes to monthly injections over one year, however, there was modest benefit of more frequent injections even over the long term.

#### 5.3.2. Pro Re Nata (PRN) and Treat and Extend (T&E) Regimens

The goal of both pro re nata (PRN) and treat and extend (T&E) dosing regimens is to effectively prevent disease progression while also minimizing the burden of treatment. The PRN approach involves regular follow-up visits, with injections only given if there is evidence that the patient’s disease is worsening. On the other hand, the T&E begins with a loading phase of injections until the patient’s condition is stable, after which the follow-up interval is gradually increased, with an injection still being given at each visit. Then, the examiner aims to find the longest interval between visits that still maintains stability of the patient’s disease.

PRN and T&E regimens for anti-VEGF injections have been studied primarily in DME, with positive results supporting their non-inferiority to fixed-interval dosing. In a meta-analysis of over 2000 patients with DME, outcomes such as BCVA and anatomical markers were similar between regimens. However, the total number of injections after 12 months of therapy was significantly lower in patients treated with a PRN regimen compared with fixed interval. There was no significant difference in number of injections between the T&E and fixed regimens for patients with DME [32].

At present there is little evidence comparing T&E or PRN approaches with fixed-interval dosing for the treatment of PDR and NPDR. However, a recent study on the open-label extension of RIDE and RISE did investigate outcomes after switching from monthly to PRN ranibizumab injection, although there was no head-to-head comparison with continued monthly injections.

An observational study on the RIDE/RISE open-label extension included 367 patients with DR and DME who had been receiving monthly 0.5 mg ranibizumab injections for at least 1 year. These patients switched from a monthly to PRN injection schedule, and were followed for changes in DRSS scores over the next 12 months. The results showed that 70% of patients maintained improvement, although the 30% of patients whose DR worsened highlights the ongoing need for management of DR even after a year of treatment with anti-VEGF therapy [33]. For patients who did experience DRS worsening, the primary explanatory factor was a history of severe NPDR that had improved to mild or moderate severity during the prior course of long-term fixed monthly treatment. In addition, patients who improved or maintained their DRSS scores typically received a higher number of as-needed injections compared to the patients who experienced DRSS worsening [34].

Another consideration for PRN and T&E regimens is choosing which indicator to use for determining whether treatment is due at a given follow-up visit. The PRIME trial, a randomized Phase 2 study in patients with NPDR and PDR, compared PRN aflibercept dosing using either DRSS score or peripheral leakage index (PLI) as the indicator for PRN treatment. When treatment was guided by DRSS score, 100% of eyes experienced DRSS worsening, while only 59% of eyes in the PLI-guided arm experienced worsening. By the same token, the mean leakage index decreased 18% in the DRSS-guided arm and decreased 55% in the PLI-guided arm [35]. Given the high proportion of patients with disease worsening using either method, these results emphasize the importance of close clinical follow-up even among patients who may have initially experienced improvement in DRSS scores. Moreover, these results highlight the need for future studies exploring different clinical indicators or other strategies for determining whether treatment is needed at follow-up when using a non-fixed interval approach such as PRN or T&E.

#### 5.3.3. Adherence

Adherence to follow-up for anti-VEGF injections is critical for the effective management of DR and maintaining visual outcomes over the long term. From a pharmacokinetics standpoint, it takes a monthly injection to maintain adequate drug levels if the goal is continuous action of anti-VEGF therapy [36]. Cessation of monthly injections may promptly result in disease worsening, even in patients who quickly showed improvement and then remained stable over the long term.

The CLARITY trial was a Phase 2b clinical trial comparing 1-year visual acuity outcomes in 232 participants with PDR randomized to either PRP or monthly aflibercept. Post hoc analysis of the CLARITY trial showed that aflibercept improved deep hemorrhages and IRMA in 75% of eyes after only three monthly injections, and the improvement was maintained at one year. However, these markers deteriorated again after the initial improvement in eyes which did not receive further injections, compared with eyes that continued injections [37], highlighting the need for continued follow-up and ongoing therapy.

Lapses in care can be detrimental, especially in patients with aggressive disease or who have been on long-term therapy. A post hoc analysis of a subset of DRCR Protocol S patients (n = 394 eyes in n = 174 participants) reported that 55% of patients had lapses in care longer than 8 weeks past a scheduled examination over a 5-year period. Lapses in care of 8 weeks or longer were associated with a statistically significant worsening of visual acuity (−2 letters from baseline) compared to those without a long lapse (who improved on average by +5 letters). Long lapses in care were also linked with increased odds of NVD and NVE [38]. Therefore, adherence to scheduled follow-up is of utmost importance for effective DR management.

## 6. Comparing Anti-VEGF versus PRP as Primary Therapy for PDR

### 6.1. Visual Outcomes and Complications: Anti-VEGF Now the Clear Front-Runner

The landmark ETDRS studies demonstrated the benefits of PRP for the treatment of PDR [5,39,40,41,42,43,44], and consequently PRP has long been regarded as the gold standard for the treatment of PDR. Although anti-VEGF therapy has emerged as a highly effective alternative, most references have maintained that outcomes are more or less equivalent when comparing the two. However, recent data has challenged this notion, favoring anti-VEGF therapy over PRP.

A recent meta-analysis of 5 studies on 632 eyes found that on average, anti-VEGF intervention in patients with PDR resulted in an additional 4 letters gained compared with PRP at 12 months, and the difference was statistically significant. The complication profile was also more favorable with anti-VEGF over PRP, with a 10% absolute risk reduction in need for future PPV and a 10% absolute risk reduction in vitreous hemorrhage rates [45].

Analysis of the CLARITY trial directly compared outcomes in patients with PDR randomized to either aflibercept or PRP in patients with PDR. Both the intention to treat (n = 221) and per protocol (n = 210) analyses showed significantly greater visual acuity improvement with aflibercept after 1 year, with a difference of about 4 letters compared to PRP [46].

DRCR Protocol S compared the long-term outcomes of eyes treated with intravitreal ranibizumab (intravitreal ranibizumab, n = 191) or PRP (PRP, n = 203) over a period of 5 years. By two years, patients treated with ranibizumab needed fewer vitrectomies and were less likely to develop CI-DME compared with patients treated with PRP [47]. At five years, DME developed in only 22% of eyes treated with intravitreal ranibizumab compared with 38% of eyes treated with PRP, and 50% of eyes in the PRP groups required a second PRP session over five years [48]. Mean change in visual acuity was also significantly better in the ranibizumab group [48], and visual field loss was significantly worse in the PRP group [49]. There was no significant difference in adverse events or patient-centered outcomes assessed by subjective questionnaires [48].

Altogether, recent data suggests that in patients with PDR, anti-VEGF is superior to PRP in terms of visual acuity benefit, DME prevention, adverse effect of visual field loss, and the need for future PPV or additional PRP.

### 6.2. Individual Patient Considerations: Follow-Up, Cost, and Demographics

When selecting the appropriate treatment, several factors must be considered apart from the primary endpoints of clinical trials. For example, patient demographic factors may tip the scale toward one therapy over another; or importantly, socioeconomic factors may potentially limit a patient’s adherence. To achieve the best outcome for an individual patient, a physician must consider all of these factors on a case-by-case basis. In the context of DR, cost of therapy and adherence have been important factors historically for choosing between PRP and anti-VEGF, while data comparing outcomes across demographics has been sparse.

A post hoc analysis of outcomes by demographic was performed after the completion of DRCR Protocol S. This analysis found that there was no patient baseline characteristic for which PRP had superior visual acuity outcomes over ranibizumab. Stratification of groups by baseline DME, VA, prior treatment history, or DR severity found no interaction between these variables and visual outcomes. However, several characteristics were associated with greater benefit of ranibizumab over PRP, including high mean arterial pressure, no previous laser, advanced PDR, and presence of NVD or NVE [50]. Similarly, post hoc analysis of CLARITY data only identified groups in which aflibercept was superior to PRP. Treatment naive patients with NVE demonstrated particularly greater benefit from aflibercept over PRP. The rate of neovascularization regression in these patients was 96% with aflibercept versus 78% with PRP, which was statistically significant [51].

On the other hand, the cost effectiveness of PRP is certainly an advantage over anti-VEGF therapy. Cost analysis using data from DRCR Protocol S trial reported that the cost per quality-adjusted life year (QALY) for ranibizumab was USD 582,268 more than PRP at 5 years, and USD 742,202 more than PRP at 10 years. This was interpreted by the authors as not cost effective in the United States, although it could become cost effective in the future if drug prices are reduced. For patients with CI-DME and vision loss, the cost was USD 65,576 greater than PRP per QALY at 5 years and USD 63,930 per QALY at 10 years, which was considered cost effective given the clinical benefits [52]. Indeed, the overall cost of ranibizumab was significantly higher than PRP. Although there are more affordable anti-VEGF agents that may yield similar benefits, the cost of anti-VEGF therapy certainly remains a limitation.

Finally, discussions of the benefits of PRP over intravitreal VEGF injections often refer to the fewer treatments needed that are long lasting with PRP, making it an attractive treatment for patients that seem unlikely to adhere to follow-up. Post hoc analysis of Phase 3 RIDE and RISE trials suggests that nearly half of patients with DR that undergo PRP will need PRP again in the future. These trials involved 577 patients with DME and either PDR or NPDR that were randomized to either intravitreal ranibizumab or sham. In patients randomized to sham injection, 40% of patients who had previously undergone PRP required additional PRP [53]. 

Even patients who have undergone PRP may benefit from anti-VEGF therapy, and this will be discussed in the following section.

## 7. Anti-VEGF as an Adjunct to PRP for PDR

Several recent studies have investigated the combination of PRP with anti-VEGF therapy. Adjunctive use of anti-VEGF in patients requiring PRP may improve outcomes where PRP alone falls short.

Post hoc analysis of RIDE and RISE trials in patients with both DR and DME suggest that there may be a role for adjuvant use of anti-VEGF therapy to prevent the need for additional PRP treatments and minimize clinical adverse ocular events. Nearly half of patients receiving sham injections and treated with PRP required at least one additional PRP treatment within two years. However, only 10% of patients treated with ranibizumab required additional PRP during the same time period, corresponding to a roughly 75% relative risk reduction in need of PRP over two years. Comparing all patients with prior PRP, those treated with sham also experienced more adverse events overall than patients treated with ranibizumab [53].

A few small trials have recently investigated a combination of anti-VEGF with PRP versus either treatment alone. These trials suggest that PRP with adjunctive anti-VEGF may serve as a sort of middle ground, yielding better clinical outcomes compared to PRP alone, but without the same level of follow-up burden as anti-VEGF monotherapy. One trial showed that a combination protocol involving photocoagulation and less frequent intravitreal bevacizumab injections showed a statistically significant decrease in the area of neovascularization with leakage compared to PRP alone or monthly intravitreal bevacizumab alone, although there was no significant difference in visual acuity, visual field, or rate of new onset DME across any of the groups. Patients in the combination group had a statistically significant reduction in the number of intravitreal bevacizumab injections compared to the intravitreal bevacizumab only group [54]. A second trial demonstrated that the use of intravitreal bevacizumab as pre-treatment 15 days before PRP resulted in significant improvement in visual acuity and regression of neovascularization at 30 days compared to PRP alone [55]. In a third trial, a single dose of adjunctive intravitreal ranibizumab with PRP was associated with improved low-frequency contrast sensitivity thresholds at one, three, and six months compared with PRP alone [56].

Currently there are no guidelines for the adjunctive use of anti-VEGF with PRP. However, the above data suggests that a reasonable approach may be a single pre-treatment prior to PRP followed by longer-interval dosing (quarterly, for instance) after PRP, although additional studies are needed to explore the best dosing interval. Taken together, these results suggest that a combination protocol might yield improved outcomes compared to PRP alone, with known benefits including greater regression of neovascularization, superior short-term visual outcomes, and preserved retinal function on electroretinography. At the same time, it would require less follow-up burden than primary anti-VEGF therapy, making this an intermediate approach.

## 8. Treatment of Severe PDR with Vitreous Hemorrhage 

In patients with severe PDR, PPV is a treatment option to restore vision and prevent additional complications. Indications for PPV are non-clearing vitreous hemorrhage, rubeosis of the iris with vitreous hemorrhage, rhegmatogenous and tractional retinal detachment, macula-involving tractional retinal detachment, and progressive fibrovascular proliferation [57,58]. Recently, PRP and anti-VEGF therapies have been studied as alternatives and adjuncts to PPV. 

### 8.1. Anti-VEGF Monotherapy as an Alternative to PPV

New data suggests that for some patients, anti-VEGF agents may delay the need for PPV or perhaps serve as an alternative to PPV for PDR with vitreous hemorrhage. In 1 RCT, 4/12 participants randomized to ranibizumab pre-treatment were able to defer PPV, while all participants in the sham group required PPV by 7 weeks. By 12 months, 3 patients treated with ranibizumab still had not required PPV. BCVA was not significantly different between groups at 12 months [59], suggesting no adverse difference in visual outcomes. These results should be cautiously interpreted given the small sample size, however, this data does suggest anti-VEGF therapy could potentially be useful for delaying PPV over at least one year in a significant proportion of patients with PDR and vitreous hemorrhage.

### 8.2. Adjunctive Use of Anti-VEGF with PPV

A wealth of evidence from recent RCTs and meta-analyses supports the preoperative adjunctive use of anti-VEGF agents. Improved intraoperative outcomes have been described consistently across several studies of anti-VEGF agents. These benefits include less intraoperative bleeding, less need for endodiathermy, reduced rates of iatrogenic retinal breaks, and significant reductions in surgical times, estimated to be in the order of at least 10 min per surgery [60,61,62].

Postoperatively, the benefits of anti-VEGF pre-treatment prior to PPV include superior visual acuity up to at least six months, shorter time to vitreous clearance, lower rates of postoperative hemorrhage, decreased likelihood of developing new CI-DME, and decreased risk of new tractional retinal detachment [62,63,64,65]. Considering the agreement across many studies, it seems that these benefits are shared across any of the anti-VEGF agents.

One important consideration is the optimal timing for anti-VEGF pre-treatment prior to PPV. One meta-analysis involving 26 RCTs and 1806 PDR patients suggested that optimal timing is 6–14 days before PPV. Empirically, this timing was associated with improved postop BCVA, decreased incidence of vitreous hemorrhage, and shorter duration of surgery [66].

### 8.3. Anti-VEGF versus Combination of PRP with PPV

Other investigations have evaluated aflibercept as monotherapy for eyes with diabetic vitreous hemorrhage compared with the combination of PRP and PPV. One study in 205 eyes found that the combination of PPV plus PRP was not superior to intravitreal aflibercept at 24 weeks in terms of visual acuity [67], although the authors speculate that this may be due to the study being underpowered. Post hoc analysis of the above study revealed faster vitreous clearance of about 4 weeks in patients treated with PRP and PPV compared with 36 weeks in patients treated with aflibercept monotherapy. In patients with visual acuity worse than 20/80, visual acuity also recovered more quickly with the combination of PPV and PRP compared with aflibercept [68].

### 8.4. Combination of Anti-VEGF with PRP versus PPV

Conversely, another RCT compared the combination of aflibercept plus PRP against PPV alone in 34 patients with PDR and vitreous hemorrhage. There was no statistically significant difference in visual acuity, but there was greater hemorrhage recurrence and longer time to clearance in eyes treated with intravitreal aflibercept + PRP compared with PPV by the final nine-month follow-up. On average, time to clearance was eight weeks in eyes treated with aflibercept and PRP, versus five days in eyes treated with PPV [69].

## 9. Conclusions

Treatments for diabetic retinopathy have evolved considerably since the landmark ETDRS and DRVS studies which demonstrated the efficacy of panretinal photocoagulation [5,39,40,41,42,43,44] and pars plana vitrectomy, [70,71,72,73,74] respectively, in patients with PDR with or without vitreous hemorrhage. Now, anti-VEGF treatment is an option to slow down the progression of DR, even in patients who have not yet developed proliferative disease. However, due to the burden of treatment with the number of injections required, it is not commonly used as a monotherapy. More commonly, PRP, PPV, and anti-VEGF agents are employed together as needed for the management of the various stages of DR.

## Figures and Tables

**Table 1:** Anti-VEGF agents for the treatment of diabetic retinopathy.

Name	Mechanism of Action	FDA Approval
Bevacizumab	149 kDa recombinant humanized monoclonal antibody comprised of two mouse antibody binding regions targeting VEGF-A, with a truncated human IgG1 heavy chain	metastatic colorectal cancer (2004), off-label for intraocular use
Ranibizumab	48 kDa recombinant monoclonal antibody fragment with one VEGF-A binding site, created from the same mouse antibody as bevacizumab, but lacking the fragment crystallizable (Fc) region and small enough to avoid Fc recycling and can more easily penetrate retinal tissue	wet AMD (2006), DME (2012), DR (2017)
Aflibercept	115 kDa recombinant soluble decoy receptor with two VEGF-binding domains, one each from VEGF-1 and VEGF-2 receptors, fused with Fc from IgG1. Traps VEGF-A, VEGF-B and PIGF and directs them to be consumed by phagocytes	wet AMD (2011), DME (2014), DR (2019)
Brolucizmab	26 kDa humanized monoclonal single-chain variable fragment. It binds VEGF-A with a single binding site in a 2:1 brolucizumab:VEGF ratio	wet AMD (2019), DME (2022), not approved for DR
Faricimab	149 kDa dual-mechanism antibody with two different antigen-binding fragment regions, one which targets VEGF and the other targeting Ang-2, connected to a single Fc domain	wet AMD (2022) and DME (2022), not approved for DR

**Table 2:** Summary of evidence for the uses and benefits of anti-VEGF agents across stages of diabetic retinopathy.

Stage of Diabetic Retinopathy (DR)	Application of Anti-VEGF Therapy	Evidence-Based Benefits	Level of Evidence
mild nonproliferative DR	none	N/A	N/A
Moderate–severe nonproliferative DR	primary monotherapy	prevention of PDR prevention of DME prevention of DRSS worsening	phase III trials: DRCR Protocol W and PANORAMA
Proliferative DR	primary monotherapy	prevention of DRSS worsening prevention of DME	phase III trial: RECOVERY
	alternative to PRP	fewer complications more ETDRS letters gained reduced risk of future hemorrhage reduced need for future vitrectomy	meta-analysis, multiple RCTs
	adjunct to PRP	better clinical outcomes compared to PRP alone reduced degree of follow-up burden compared with anti-VEGF therapy alone prevention of the need for additional PRP treatments, reduced adverse ocular events	post hoc analyses of phase III RIDE and RISE trials, several small trials
	adjunct to pars plana vitrectomy	less intraoperative bleeding and need for endodiathermy reduced rates of iatrogenic retinal breaks reductions in surgical times superior visual acuity up to at least 6 months shorter time to vitreous clearance lower rates of postoperative hemorrhage decreased likelihood of developing new CI-DME decreased risk of new tractional retinal detachment	meta-analysis, multiple RCTs

## Data Availability

Not applicable.

## Abbreviations

BCVA	best corrected visual acuity
DR	diabetic retinopathy
DRSS	diabetic retinopathy severity scale
DME	diabetic macular edema
Fc	fragment crystallizable
NPDR	nonproliferative diabetic retinopathy
NVD	neovascularization of the disc
NVE	neovascularization elsewhere
NVI	neovascularization of the iris
RCT	randomized controlled trial
PDR	proliferative diabetic retinopathy
PRP	panretinal photocoagulation
PPV	pars plana vitrectomy
PRN	pro re nata
T&E	treat and extend
VEGF	vascular endothelial growth factor

## References

[1] Teo Z.L., Tham Y.C., Yu M., Chee M.L., Rim T.H., Cheung N., Bikbov M.M., Wang Y.X., Tang Y., Lu Y. (2021). Global Prevalence of Diabetic Retinopathy and Projection of Burden through 2045: Systematic Review and Meta-analysis. Ophthalmology.

[2] Kim L.A., Khalaf H.S., Lim J.I. (2022). Neurodegeneration in Diabetic Retinopathy. Secondary Neurodegeneration in Diabetic Retinopathy. https://eyewiki.aao.org/Neurodegeneration_in_Diabetic_Retinopathy.

[3] Klein R., Klein B.E., Moss S.E., Linton K.L. (1992). The Beaver Dam Eye Study. Retinopathy in adults with newly discovered and previously diagnosed diabetes mellitus. Ophthalmology.

[4] Aiello L.M., Berrocal J., Davis M.D., Ederer F., Goldberg M.F., Harris J.E., Klimt C.R., Knatterud G.L., Margherio R.R., McLean E.N. (1973). Editorial: The diabetic retinopathy study. Arch. Ophthalmol..

[5] Early Treatment Diabetic Retinopathy Study Research Group (1991). Early Photocoagulation for Diabetic Retinopathy. Ophthalmology.

[6] Fogli S., Del Re M., Rofi E., Posarelli C., Figus M., Danesi R. (2018). Clinical pharmacology of intravitreal anti-VEGF drugs. Eye.

[7] Pignatelli F., Niro A., Passidomo F., Addabbo G. (2021). Molecular structure, pharmacokinetics and clinical evidence of brolucizumab: A narrative review. Ann. Eye Sci..

[8] Falavarjani K.G., Nguyen Q.D. (2013). Adverse events and complications associated with intravitreal injection of anti-VEGF agents: A review of literature. Eye.

[9] Shah S.M., Khanna C.L., Yamanuha J., Bakri S.J. (2020). “Glaucomatous fields” after monthly intravitreal injections: Normal tension glaucoma or a mimicker. Am. J. Ophthalmol. Case Rep..

[10] Thulliez M., Angoulvant D., Pisella P.J., Bejan-Angoulvant T. (2018). Overview of Systematic Reviews and Meta-analyses on Systemic Adverse Events Associated with Intravitreal Anti-Vascular Endothelial Growth Factor Medication Use. JAMA Ophthalmol..

[11] Atchison E.A., Omar A.F., Iezzi R., Barkmeier A.J., Bakri S.J. (2017). Outcomes of an Intravitreal Injection Clinic. Retina.

[12] Dalvin L.A., Starr M.R., AbouChehade J.E., Damento G.M., Garcia M., Shah S.M., Hodge D.O., Meissner I., Bakri S.J., Iezzi R. (2019). Association of Intravitreal Anti-Vascular Endothelial Growth Factor Therapy with Risk of Stroke, Myocardial Infarction, and Death in Patients with Exudative Age-Related Macular Degeneration. JAMA Ophthalmol..

[13] Mansukhani S.A., Barkmeier A.J., Bakri S.J., Iezzi R., Pulido J.S., Khanna C.L., Bennett J.R., Hodge D.O., Sit A.J. (2018). The Risk of Primary Open-Angle Glaucoma Following Vitreoretinal Surgery-A Population-based Study. Am. J. Ophthalmol..

[14] Shah S.M., Boopathiraj N., Starr M.R., Dalvin L.A., AbouChehade J., Damento G., Garcia M.D., Hodge D.O., Bakri S.J., Sit A.J. (2022). Risk, Prevalence, and Progression of Glaucoma in Eyes with Age-Related Macular Degeneration Treated with Intravitreal Anti-Vascular Endothelial Growth Factor Injections. Am. J. Ophthalmol..

[15] Starr M.R., Dalvin L.A., AbouChehade J.E., Damento G.M., Garcia M.D., Shah S.M., Hodge D.O., Meissner I., Iezzi R., Bakri S.J. (2019). Classification of Strokes in Patients Receiving Intravitreal Anti-Vascular Endothelial Growth Factor. Ophthalmic Surg. Lasers Imaging Retin..

[16] Reibaldi M., Fallico M., Avitabile T., Marolo P., Parisi G., Cennamo G., Furino C., Lucenteforte E., Virgili G. (2022). Frequency of Intravitreal Anti-VEGF Injections and Risk of Death: A Systematic Review with Meta-analysis. Ophthalmol. Retin..

[17] Rebeiz A.G., Mahfoud Z., Abdul Fattah M., Saad A., Safar A., Bashshur Z.F. (2020). Change in cardiac troponin T level after intravitreal anti-vascular endothelial growth factor treatment: Prospective pilot study. Eur. J. Ophthalmol..

[18] Green M., Tien T., Ness S. (2020). Predictors of Lost to Follow-Up in Patients Being Treated for Proliferative Diabetic Retinopathy. Am. J. Ophthalmol..

[19] Obeid A., Su D., Patel S.N., Uhr J.H., Borkar D., Gao X., Fineman M.S., Regillo C.D., Maguire J.I., Garg S.J. (2019). Outcomes of Eyes Lost to Follow-up with Proliferative Diabetic Retinopathy That Received Panretinal Photocoagulation versus Intravitreal Anti-Vascular Endothelial Growth Factor. Ophthalmology.

[20] Bressler N.M., Beaulieu W.T., Bressler S.B., Glassman A.R., Melia B.M., Jampol L.M., Jhaveri C.D., Salehi-Had H., Velez G., Sun J.K. (2020). Anti-Vascular Endothelial Growth Factor Therapy and Risk Of Traction Retinal Detachment in Eyes with Proliferative Diabetic Retinopathy: Pooled Analysis of Five DRCR Retina Network Randomized Clinical Trials. Retina.

[21] Reddy S.V., Husain D. (2018). Panretinal Photocoagulation: A Review of Complications. Semin. Ophthalmol..

[22] Stein J.D., Zacks D.N., Grossman D., Grabe H., Johnson M.W., Sloan F.A. (2009). Adverse events after pars plana vitrectomy among medicare beneficiaries. Arch. Ophthalmol..

[23] Maturi R.K., Glassman A.R., Josic K., Antoszyk A.N., Blodi B.A., Jampol L.M., Marcus D.M., Martin D.F., Melia M., Salehi-Had H. (2021). Effect of Intravitreous Anti-Vascular Endothelial Growth Factor vs. Sham Treatment for Prevention of Vision-Threatening Complications of Diabetic Retinopathy: The Protocol W Randomized Clinical Trial. JAMA Ophthalmol..

[24] Maturi R.K., Glassman A.R., Josic K., Baker C.W., Gerstenblith A.T., Jampol L.M., Meleth A., Martin D.F., Melia M., Punjabi O.S. (2023). Four-Year Visual Outcomes in the Protocol W Randomized Trial of Intravitreous Aflibercept for Prevention of Vision-Threatening Complications of Diabetic Retinopathy. JAMA.

[25] Brown D.M., Wykoff C.C., Boyer D., Heier J.S., Clark W.L., Emanuelli A., Higgins P.M., Singer M., Weinreich D.M., Yancopoulos G.D. (2021). Evaluation of Intravitreal Aflibercept for the Treatment of Severe Nonproliferative Diabetic Retinopathy: Results from the PANORAMA Randomized Clinical Trial. JAMA Ophthalmol..

[26] Wykoff C.C., Nittala M.G., Zhou B., Fan W., Velaga S.B., Lampen S.I.R., Rusakevich A.M., Ehlers J.P., Babiuch A., Brown D.M. (2019). Intravitreal Aflibercept for Retinal Nonperfusion in Proliferative Diabetic Retinopathy: Outcomes from the Randomized RECOVERY Trial. Ophthalmol. Retin..

[27] Wykoff C.C., Nittala M.G., Villanueva Boone C., Yu H.J., Fan W., Velaga S.B., Ehlers J.P., Ip M.S., Sadda S.R., Group R.S. (2022). Final Outcomes from the Randomized RECOVERY Trial of Aflibercept for Retinal Nonperfusion in Proliferative Diabetic Retinopathy. Ophthalmol. Retin..

[28] Alagorie A.R., Velaga S., Nittala M.G., Yu H.J., Wykoff C.C., Sadda S.R. (2021). Effect of Aflibercept on Diabetic Retinopathy Severity and Visual Function in the RECOVERY Study for Proliferative Diabetic Retinopathy. Ophthalmol. Retin..

[29] Babiuch A.S., Wykoff C.C., Srivastava S.K., Talcott K., Zhou B., Hach J., Hu M., Reese J.L., Ehlers J.P. (2020). Retinal Leakage Index Dynamics on Ultra-Widefield Fluorescein Angiography in Eyes Treated with Intravitreal Aflibercept for Proliferative Diabetic Retinopathy in the Recovery Study. Retina.

[30] Fan W., Nittala M.G., Wykoff C.C., Brown D.M., Uji A., Hemert J.V., Fleming A., Robertson G., Sadda S.R., Ip M. (2022). New Biomarker Quantifying the Effect Of Anti-Vegf Therapy in Eyes with Proliferative Diabetic Retinopathy on Ultrawide Field Fluorescein Angiography: Recovery Study. Retina.

[31] Babiuch A., Wykoff C.C., Hach J., Srivastava S., Talcott K.E., Yu H.J., Nittala M., Sadda S., Ip M.S., Le T. (2021). Longitudinal panretinal microaneurysm dynamics on ultra-widefield fluorescein angiography in eyes treated with intravitreal aflibercept for proliferative diabetic retinopathy in the recovery study. Br. J. Ophthalmol..

[32] Sarohia G.S., Nanji K., Khan M., Khalid M.F., Rosenberg D., Deonarain D.M., Phillips M.R., Thabane L., Kaiser P.K., Garg S.J. (2022). Treat-and-extend versus alternate dosing strategies with anti-vascular endothelial growth factor agents to treat center involving diabetic macular edema: A systematic review and meta-analysis of 2346 eyes. Surv. Ophthalmol..

[33] Sun J.K., Wang P.W., Taylor S., Haskova Z. (2019). Durability of Diabetic Retinopathy Improvement with As-Needed Ranibizumab: Open-Label Extension of RIDE and RISE Studies. Ophthalmology.

[34] Goldberg R.A., Hill L., Davis T., Stoilov I. (2022). Effect of less aggressive treatment on diabetic retinopathy severity scale scores: Analyses of the RIDE and RISE open-label extension. BMJ Open Ophthalmol..

[35] Yu H.J., Ehlers J.P., Sevgi D.D., Hach J., O’Connell M., Reese J.L., Srivastava S.K., Wykoff C.C. (2021). Real-Time Photographic- and Fluorescein Angiographic-Guided Management of Diabetic Retinopathy: Randomized PRIME Trial Outcomes. Am. J. Ophthalmol..

[36] Xu M., Fan R., Fan X., Shao Y., Li X. (2022). Progress and Challenges of Anti-VEGF Agents and Their Sustained-Release Strategies for Retinal Angiogenesis. Drug. Des. Devel Ther..

[37] Pearce E., Chong V., Sivaprasad S. (2020). Aflibercept Reduces Retinal Hemorrhages and Intravitreal Microvascular Abnormalities but Not Venous Beading: Secondary Analysis of the CLARITY Study. Ophthalmol. Retin..

[38] Maguire M.G., Liu D., Bressler S.B., Friedman S.M., Melia M., Stockdale C.R., Glassman A.R., Sun J.K., Network D.R. (2021). Lapses in Care among Patients Assigned to Ranibizumab for Proliferative Diabetic Retinopathy: A Post Hoc Analysis of a Randomized Clinical Trial. JAMA Ophthalmol..

[39] Early Treatment Diabetic Retinopathy Study Research Group (1991). Classification of diabetic retinopathy from fluorescein angiograms. ETDRS report number 11. Ophthalmology.

[40] (1991). Early Treatment Diabetic Retinopathy Study design and baseline patient characteristics. ETDRS report number 7. Ophthalmology.

[41] Early Treatment Diabetic Retinopathy Study Research Group (1991). Fluorescein angiographic risk factors for progression of diabetic retinopathy. ETDRS report number 13. Ophthalmology.

[42] Early Treatment Diabetic Retinopathy Study Research Group (1991). Fundus photographic risk factors for progression of diabetic retinopathy. ETDRS report number 12. Ophthalmology.

[43] Early Treatment Diabetic Retinopathy Study Research Group (1991). Grading diabetic retinopathy from stereoscopic color fundus photographs—An extension of the modified Airlie House classification. ETDRS report number 10. Ophthalmology.

[44] Flynn H.W., Chew E.Y., Simons B.D., Barton F.B., Remaley N.A., Ferris F.L. (1992). Pars plana vitrectomy in the Early Treatment Diabetic Retinopathy Study. ETDRS report number 17. The Early Treatment Diabetic Retinopathy Study Research Group. Ophthalmology.

[45] Yates W.B., Mammo Z., Simunovic M.P. (2021). Intravitreal anti-vascular endothelial growth factor versus panretinal LASER photocoagulation for proliferative diabetic retinopathy: A systematic review and meta-analysis. Can. J. Ophthalmol..

[46] Sivaprasad S., Hykin P., Prevost A.T., Vasconcelos J., Riddell A., Ramu J., Murphy C., Kelly J., Edwards R.T., Yeo S.T. (2018). Intravitreal aflibercept compared with panretinal photocoagulation for proliferative diabetic retinopathy: The CLARITY non-inferiority RCT. Effic. Mech. Eval..

[47] Sun J.K., Glassman A.R., Beaulieu W.T., Stockdale C.R., Bressler N.M., Flaxel C., Gross J.G., Shami M., Jampol L.M., Diabetic Retinopathy Clinical Research Network (2019). Rationale and Application of the Protocol S Anti-Vascular Endothelial Growth Factor Algorithm for Proliferative Diabetic Retinopathy. Ophthalmology.

[48] Gross J.G., Glassman A.R., Liu D., Sun J.K., Antoszyk A.N., Baker C.W., Bressler N.M., Elman M.J., Ferris F.L., Gardner T.W. (2018). Five-Year Outcomes of Panretinal Photocoagulation vs. Intravitreous Ranibizumab for Proliferative Diabetic Retinopathy: A Randomized Clinical Trial. JAMA Ophthalmol..

[49] Maguire M.G., Liu D., Glassman A.R., Jampol L.M., Johnson C.A., Baker C.W., Bressler N.M., Gardner T.W., Pieramici D., Stockdale C.R. (2020). Visual Field Changes over 5 Years in Patients Treated with Panretinal Photocoagulation or Ranibizumab for Proliferative Diabetic Retinopathy. JAMA Ophthalmol..

[50] Bressler S.B., Beaulieu W.T., Glassman A.R., Gross J.G., Melia M., Chen E., Pavlica M.R., Jampol L.M., Diabetic Retinopathy Clinical Research Network (2019). Photocoagulation versus Ranibizumab for Proliferative Diabetic Retinopathy: Should Baseline Characteristics Affect Choice of Treatment?. Retina.

[51] Halim S., Nugawela M., Chakravarthy U., Peto T., Madhusudhan S., Lenfestey P., Hamill B., Zheng Y., Parry D., Nicholson L. (2021). Topographical Response of Retinal Neovascularization to Aflibercept or Panretinal Photocoagulation in Proliferative Diabetic Retinopathy: Post Hoc Analysis of the CLARITY Randomized Clinical Trial. JAMA Ophthalmol..

[52] Hutton D.W., Stein J.D., Glassman A.R., Bressler N.M., Jampol L.M., Sun J.K., Network D.R. (2019). Five-Year Cost-effectiveness of Intravitreous Ranibizumab Therapy vs. Panretinal Photocoagulation for Treating Proliferative Diabetic Retinopathy: A Secondary Analysis of a Randomized Clinical Trial. JAMA Ophthalmol..

[53] Gonzalez V.H., Wang P.W., Ruiz C.Q. (2021). Panretinal Photocoagulation for Diabetic Retinopathy in the RIDE and RISE Trials: Not “1 and Done”. Ophthalmology.

[54] Shahraki T., Arabi A., Nourinia R., Beheshtizadeh N.F., Entezari M., Nikkhah H., Karimi S., Ramezani A. (2022). Panretinal Photocoagulation versus Intravitreal Bevacizumab versus a Proposed Modified Combination Therapy for Treatment of Proliferative Diabetic Retinopathy: A Randomized Three-Arm Clinical Trial (CTPDR Study). Retina.

[55] Ali W., Abbasi K.Z., Raza A. (2018). Panretinal Photocoagulation Plus Intravitreal Bevacizumab versus Panretinal Photocoagulation Alone for Proliferative Diabetic Retinopathy. J. Coll. Physicians Surg. Pak..

[56] Rentiya Z.S., Ferraz D.A., Hutnik R., Bae J., Machado C.G., Mucciolli C., Motta A., Ribeiro L.Z., Guan Z., Preti R.C. (2022). Evaluation of contrast sensitivity in non-high-risk proliferative diabetic retinopathy treated with panretinal photocoagulation with and without intravitreal injections of ranibizumab. Arq. Bras. Oftalmol..

[57] De Maria M., Panchal B., Coassin M. (2018). Update on indications for diabetic vitrectomy and management of complications. Ann. Eye Sci..

[58] Naresh Shah P.P., Shanmugam M.K., Mishra D. (2022). Vitrectomy in Diabetic Retinopathy. Diabetic Eye Disease—From Therapeutic Pipeline to the Real World.

[59] Petrarca R., Soare C., Wong R., Desai R., Neffendorf J., Simpson A., Jackson T.L. (2020). Intravitreal ranibizumab for persistent diabetic vitreous haemorrhage: A randomised, double-masked, placebo-controlled feasibility study. Acta Ophthalmol..

[60] Arevalo J.F., Lasave A.F., Kozak I., Al Rashaed S., Al Kahtani E., Maia M., Farah M.E., Cutolo C., Brito M., Osorio C. (2019). Preoperative Bevacizumab for Tractional Retinal Detachment in Proliferative Diabetic Retinopathy: A Prospective Randomized Clinical Trial. Am. J. Ophthalmol..

[61] Chen G.H., Tzekov R., Mao S.H., Tong Y.H., Jiang F.Z., Li W.S. (2022). Intravitreal conbercept as an adjuvant in vitrectomy for proliferative diabetic retinopathy: A meta-analysis of randomised controlled trials. Eye.

[62] Chen H.J., Wang C.G., Dou H.L., Feng X.F., Xu Y.M., Ma Z.Z. (2020). Effect of intravitreal ranibizumab pretreatment on vitrectomy in young patients with proliferative diabetic retinopathy. Ann. Palliat. Med..

[63] Chakraborty D., Maiti A., Kelkar A., Sengupta S., Roy S., Bolisetty M., Kothari A., Majumder J. (2021). Outcomes of preoperative bevacizumab in diabetics with nonclearing vitreous hemorrhage without tractional detachment—A quasi-randomized retrospective study. Indian. J. Ophthalmol..

[64] Li S., Yang Y., Zou J., Zeng J., Ding C. (2022). The efficacy and safety of intravitreal injection of Ranibizumab as pre-treatment for vitrectomy in proliferative diabetic retinopathy with vitreous hemorrhage. BMC Ophthalmol..

[65] Perez-Argandona E., Verdaguer J., Zacharias S., Gonzalez R. (2019). Preoperative intravitreal bevacizumab for proliferative diabetic retinopathy patients undergoing vitrectomy—First update. Medwave.

[66] Wang D.Y., Zhao X.Y., Zhang W.F., Meng L.H., Chen Y.X. (2020). Perioperative anti-vascular endothelial growth factor agents treatment in patients undergoing vitrectomy for complicated proliferative diabetic retinopathy: A network meta-analysis. Sci. Rep..

[67] Antoszyk A.N., Glassman A.R., Beaulieu W.T., Jampol L.M., Jhaveri C.D., Punjabi O.S., Salehi-Had H., Wells J.A., Maguire M.G., Stockdale C.R. (2020). Effect of Intravitreous Aflibercept vs. Vitrectomy with Panretinal Photocoagulation on Visual Acuity in Patients with Vitreous Hemorrhage from Proliferative Diabetic Retinopathy: A Randomized Clinical Trial. JAMA.

[68] Glassman A.R., Beaulieu W.T., Maguire M.G., Antoszyk A.N., Chow C.C., Elman M.J., Jampol L.M., Salehi-Had H., Sun J.K., Network D.R. (2021). Visual Acuity, Vitreous Hemorrhage, and Other Ocular Outcomes after Vitrectomy vs. Aflibercept for Vitreous Hemorrhage Due to Diabetic Retinopathy: A Secondary Analysis of a Randomized Clinical Trial. JAMA Ophthalmol..

[69] Abd Elhamid A.H., Mohamed A., Khattab A.M. (2020). Intravitreal Aflibercept injection with Panretinal photocoagulation versus early Vitrectomy for diabetic vitreous hemorrhage: Randomized clinical trial. BMC Ophthalmol..

[70] The Diabetic Retinopathy Vitrectomy Study Research Group (1988). Early vitrectomy for severe proliferative diabetic retinopathy in eyes with useful vision. Clinical application of results of a randomized trial—Diabetic Retinopathy Vitrectomy Study Report 4. Ophthalmology.

[71] The Diabetic Retinopathy Vitrectomy Study Research Group (1988). Early vitrectomy for severe proliferative diabetic retinopathy in eyes with useful vision. Results of a randomized trial—Diabetic Retinopathy Vitrectomy Study Report 3. Ophthalmology.

[72] (1990). Early vitrectomy for severe vitreous hemorrhage in diabetic retinopathy. Four-year results of a randomized trial: Diabetic Retinopathy Vitrectomy Study Report 5. Arch. Ophthalmol..

[73] The Diabetic Retinopathy Vitrectomy Study Research Group (1985). Early vitrectomy for severe vitreous hemorrhage in diabetic retinopathy. Two-year results of a randomized trial. Diabetic Retinopathy Vitrectomy Study report 2. Arch. Ophthalmol..

[74] (1985). Two-year course of visual acuity in severe proliferative diabetic retinopathy with conventional management. Diabetic Retinopathy Vitrectomy Study (DRVS) report #1. Ophthalmology.

